# Meningitis caused by *Chromobacterium haemolyticum* suspected to be derived from a canal in Japan: a case report

**DOI:** 10.1186/s13256-023-03913-1

**Published:** 2023-04-30

**Authors:** Kumi Iwamoto, Masaki Yamamoto, Atsushi Yamamoto, Toshi Sai, Toshiko Mukai, Noriko Miura, Haruhisa Kozono, Shigeki Onishi, Seiko Ohno, Nobuki Iwamoto, Yasufumi Matsumura, Miki Nagao, Yoji Urata

**Affiliations:** 1 Department of Medical Laboratory, Japanese Red Cross Kyoto Daiichi Hospital, Kyoto, Japan; 2 Department of Clinical Laboratory Medicine, Kyoto University Graduate School of Medicine, 54 Shogoin-Kawaharacho, Sakyo-Ku, Kyoto, 6068507 Japan; 3 Department of Cerebral Neurology and Stroke, Japanese Red Cross Kyoto Daiichi Hospital, Kyoto, Japan; 4 Department of Infection Control, Japanese Red Cross Kyoto Daiichi Hospital, Kyoto, Japan

**Keywords:** Case report, *Chromobacterium haemolyticum*, *Chromobacterium violaceum*, Meningitis, Misidentification

## Abstract

**Background:**

The genus *Chromobacterium*, of which 12 species have been recognized, comprises bacteria that reside in tropical and subtropical environments. Of these species, *Chromobacterium violaceum* and *Chromobacterium haemolyticum* are known to cause infections in humans. There have been few reports of infections caused by *Chromobacterium haemolyticum*.

**Case presentation:**

*Chromobacterium haemolyticum* was detected in spinal fluid and blood samples isolated from a 73-year-old Japanese male patient who fell into a canal in Kyoto City, Japan and developed bacteremia and meningitis. Although meropenem and vancomycin were administered, this patient died 9 days after admission. Although the infection was misidentified as being caused by *Chromobacterium violaceum* by conventional identification methods, average nucleotide identity analysis revealed that the causative pathogen was *Chromobacterium haemolyticum*. The same bacteria were also detected in the canal in which the accident occurred. Phylogenetic analysis of the strain isolated from the patient and the strain isolated from the canal suggested that the two strains were very closely related.

**Conclusions:**

*Chromobacterium haemolyticum* can be misidentified as *Chromobacterium violaceum* by conventional identification methods and tends to be more resistant to β-lactams than *Chromobacterium violaceum*. Pigment production and β-hemolysis on blood sheep agar can provide clues for the early identification of *Chromobacterium haemolyticum*.

## Background

The genus *Chromobacterium* comprises bacteria that reside in tropical and subtropical environments, among which *Chromobacterium violaceum* and *Chromobacterium haemolyticum* are known to cause infections in humans [1]. *C. haemolyticum* is a facultative anaerobic Gram-negative rod that is known for producing hemolysin and showing a distinct β-hemolytic zone around the colony on sheep blood agar medium [2]. A few cases of infectious diseases, including pneumonia, bacteremia, necrotizing fasciitis, and pediatric proctitis, caused by *C. haemolyticum* have been reported [1–6]. While the worldwide geographic distribution of *C. violaceum* has been reported to be concentrated in tropical and subtropical regions [7], that of *C. haemolyticum* is not well known. In this study, *C. haemolyticum* was detected in spinal fluid specimens isolated from a patient who developed meningitis after falling into a canal in Kyoto City, Japan, which is a temperate region.

## Case presentation

**Fig. 1:**
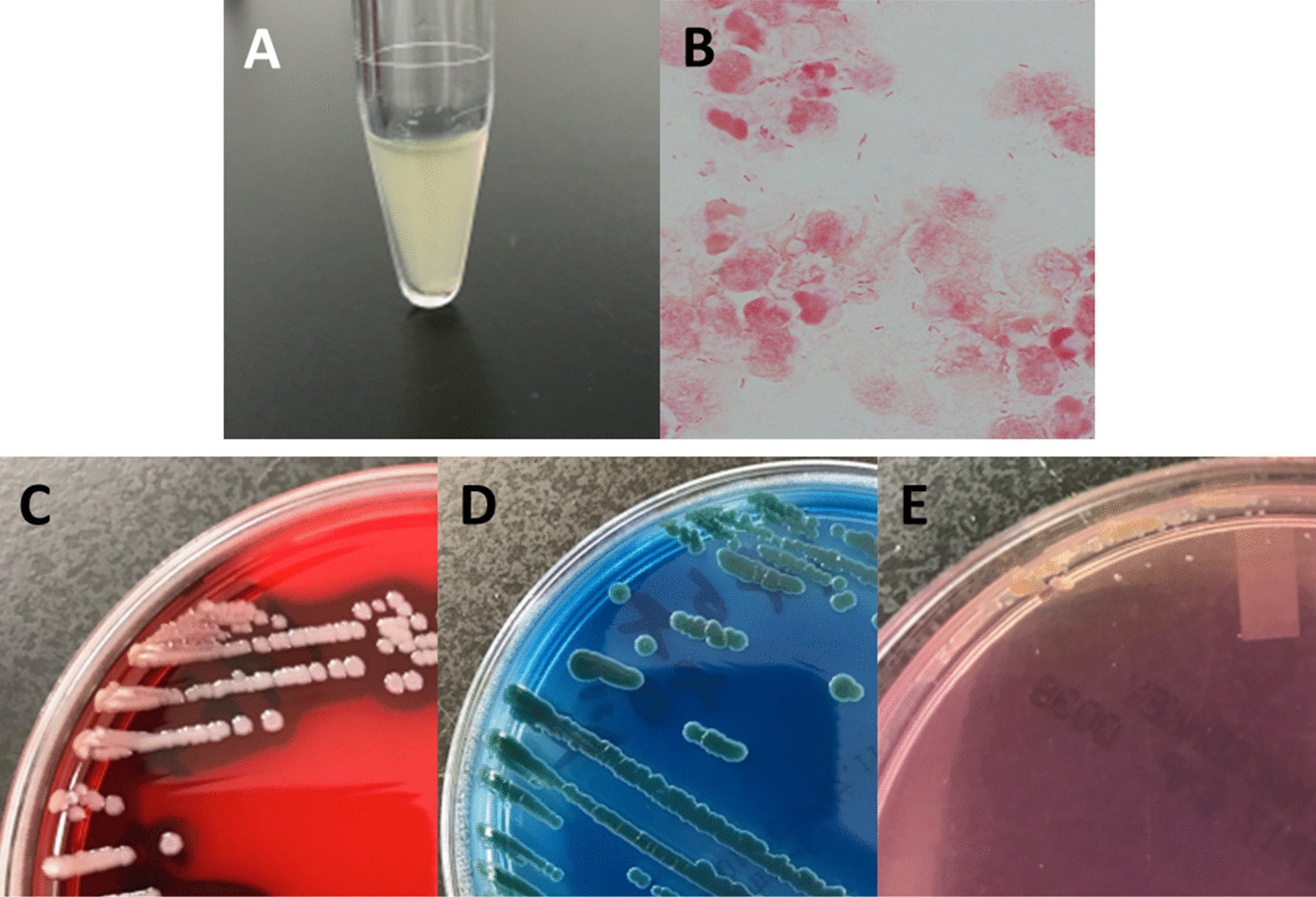
Microbiological results. **A** The spinal fluid specimen appeared cloudy. **B** Numerous Gram-negative rods were present in the spinal fluid along with inflammatory findings with increasing numbers of neutrophils (Gram stain, ×1000 original magnification). **C** Results of assessments of *Chromobacterium haemolyticum* colonies on TSA II 5% sheep blood agar after a 48-h culture at 37 °C, showing yellow colonies with clear β-hemolysis, **D** Presence of lactose nondegradable colonies on BTB lactose-added agar, **E** Presence of transparent microcolonies on MacConkey II agar

A 73-year-old Japanese male patient fell into a canal in Kyoto City in the summer of 2017 and was transported to an emergency department. He had a medical history of alcoholic hepatitis, hypertension, and hyperuricemia. He had no significant family medical history. He was an ex-smoker (stopped smoking 10 years prior) and had a history of alcohol consumption, drinking 50 g of alcohol daily. He had a 10 cm contusion wound on the back of the head and multiple fractures of the spinous process of the cervical vertebrae. On examination, his temperature was 36.1 °C, heart rate was 88 beats per minute, blood pressure was 170/96 mmHg, and respiratory rate was 18 breaths per minute. On admission, the physical and neurological examination revealed no abnormalities except for neck pain or the pre-existing mild ptosis in the left eye. He vomited on day 5 and had diminished consciousness on day 6. Computed tomography (CT) scan of the head revealed no obvious abnormalities, and a spinal tap was performed. Laboratory findings on day 6 of admission are presented in Table 1. The analysis revealed findings of bacterial meningitis with increased numbers of neutrophils, decreased glucose levels, and increased protein levels. The spinal fluid specimen exhibited a cloudy yellow appearance, and Gram staining revealed numerous Gram-negative rods along with inflammatory findings and increasing neutrophil levels (Fig. 1A, B). On the same day, blood culture samples were collected with two sets of aerobic bottles (BacT/ALERT SA; BioMerieux, Marcy l’Etoile, France) and anaerobic bottles (BacT/ALERT SN; BioMerieux) and tested by BacT/ALERT 3D (BioMerieux). Meropenem (2 g q8h) and vancomycin (1.25 g q8h) administration by infusion was initiated, and vancomycin administration was stopped after 3 days. Gram-negative rods were detected in all blood culture bottles, which all exhibited the same appearance as the spinal fluid. Cultures of spinal fluid and subcultures from the blood culture bottle were performed on sheep blood agar (Becton Dickinson: BD, Franklin Lakes, NJ, USA), Drigalski modified agar (Eiken Chemical Co., Ltd., Tokyo, Japan), and McConkey II agar medium (BD) at 37 °C for 48 hours (Fig. 1C, D). From each specimen, yellow colonies with β-hemolytic rings developed on blood agar, and green colonies developed on Drigalski modified agar. Small colonies developed on McConkey II agar medium (BD) (Fig. 1E). These colonies were nonproductive pigments. They were identified with the MicroScan WalkAway 96Plus (Beckman Coulter, Brea, CA, USA), resulting in a biotype of 40002007 with a 99.65% probability of being *C. violaceum* (the second candidate was *Vibrio damsel* with a probability of 0.35%). Identification testing by BBL CRYSTAL/NF (BD, version 5,4,2) resulted in the identification of biotype 3001310113, with only *C. violaceum* as a candidate for identification; MALDI Biotyper (Bruker Daltonics Inc., Billerica, MA, USA) yielded no peaks, and microbes were not identifiable. The main biochemical characteristics of this strain were oxidase (+), indole (−), mannose (−), and citric acid (+). Susceptibility testing was performed using the EN1J panel of the MicroScan WalkAway 96Plus. The results showed high minimum inhibitory concentration (MIC) values for penicillins, first- and second-generation cephalosporins, and ceftriaxone (Table 2). On day 8, the patient had frequent generalized convulsions, and he died on day 9. An autopsy was performed, and infiltration of inflammatory cells was detected in the subarachnoid space, which supported the diagnosis of meningitis.

**Table 1:** Laboratory findings on day 6 of admission

Hematology		Biochemistry	
WBC	26,950/μL	TP	7.4 g/dL
RBC	4.25 × 10^6^/μL	ALB	3.7 g/dL
Hb	14.7 mg/dL	AST	35 IU/L
Ht	41.00%	ALT	39 IU/L
Plt	21.3 × 10^4^/μL	LDH	309 IU/L
		ALP	195 IU/L
		T-Bil	1.2 mg/dL
Cerebrospinal fluid		D-Bil	0.1 mg/dL
TP	6.72 g/dL	CK	494 IU/L
Glu	< 10 mg/dL	AMY	73 IU/L
Number of cells	47,104/mm^3^	BUN	23 mg/dL
	(mostly neutrophils)	CRE	0.69 mg/dL
		Ca	9.5 mg/dL
		Na	41 mEq/L
		Cl	107 mEq/L
		K	3.7 mEq/L
		Glu	191 mg/dL
		CRP	30.36 mg/dL

*WBC* white blood cell; *RBC* red blood cell; *Hb* hemoglobin; *Ht* hematocrit, *Plt* platelet; *TP* total protein; *Glu* glucose; *ALB* albumin; *AST* aspartate aminotransferase; *ALT* alanine aminotransferase; *LDH* lactate dehydrogenase; *ALP* alkaline phosphatase; *T-Bil* total bilirubin; *D-Bil* direct bilirubin; *CK* creatine kinase; *AMY* amylase; *BUN* blood urea nitrogen; *CRE* creatinine; *CRP* C-reactive protein

**Table 2:** Results of susceptibility testing for *C. haemolyticum*

Antimicrobial agent	MIC (μg/mL)
Ampicillin/sulbactam	> 16
Piperacillin	> 64
Piperacillin/tazobactam	≤ 1
Cefazolin	> 16
Cefotiam	> 16
Cefotaxime	≤ 1
Ceftriaxone	> 2
Ceftazidime	≤ 4
Sulbactam/cefoperazone	≤ 16
Cefepime	≤ 2
Cefmetazole	≤ 8
Flomoxef	≤ 8
Aztreonam	≤ 4
Imipenem	2
Meropenem	≤ 1
Amikacin	> 32
Gentamycin	8
Minocycline	≤ 2
Levofloxacin	≤ 0.5
Trimethoprim/sulfamethoxazole	≤ 2

*MIC* minimum inhibitory concentration

A water culture survey was conducted at the site of the accidental fall, and the canal was approximately 1 m wide and 10 cm deep. Water samples were taken from eight locations over a 10 m stretch, and 50 µL of each sample was cultured on BTB lactose-supplemented agar medium. As a result, 20–30 colony-forming units (CFUs) of colony growth were suspected to comprise *Chromobacterium* spp. and were observed in samples from all locations, and these were identified as *C. violaceum* by MicroScan WalkAway 96Plus.

Whole-genome sequencing was performed with 151-cycle paired-end reads on an Illumina NextSeq 550 platform (Illumina, San Diego, CA, USA). All of the Illumina read data obtained from clinical and environmental isolates were deposited into the National Center for Biotechnology Information Sequence Read Archive database, SRA (accession numbers SRR20306466 and SRR20306467). Average nucleotide identity (ANI) analysis using JSpecies V1.2.1 [8] showed that the concordance rates between the clinical isolate strain and *C. violaceum* (ATCC12472), *C. haemolyticum* (DSM19808), and the strain from the canal were 85.42%, 96.49%, and 99.89%, respectively, and these clinical and environmental strains, each identified as *C. violaceum* by conventional methods, were *C. haemolyticum*. Then, phylogenetic analysis was performed using kSNP v3.1 with default parameters (*k*-mer = 21) [9]. There were only three single nucleotide polymorphisms (SNPs) between the strain from the patient and the strain from the canal. This finding indicates that both analyzed strains may have been derived from the same strain.

## Discussion and conclusions

We report a fatal case of bacterial meningitis caused by *C. haemolyticum* acquired from a canal. *C. haemolyticum* was isolated from both this patient and the canal water, and the isolates were genomically closely related. This rare case revealed evidence supporting the development of severe meningitis accompanied by the invasion of environmental bacteria into the human body.

Twelve species of *Chromobacterium* spp. have been recognized [10]. Of these species, only *C. violaceum* and *C. haemolyticum* have been reported to infect humans [4]. *C. violaceum* is characterized by producing a purple pigment called violacein, and a high mortality rate of 53% resulting from infections caused by *C. violaceum* has been reported [7]. Alisjahbana *et al.* reviewed 132 cases of disease caused by *C. violaceum* in humans reported between 1953 and 2021, and the results showed that the organism is distributed worldwide in warm regions between 35° N and 35° S latitudes and causes human infections [11]. However, only six cases of infections caused by *C. haemolyticum* were reported, three of which were in Japan [1–6]. Although there have been six cases of infection caused by *C. haemolyticum*, this is the first case of meningitis caused by this bacterium that we know of (Table 3). In two of six cases observed in Japan, phylogenetic analysis was conducted using pulsed-field gel electrophoresis or whole-genome sequencing to compare the patient-derived strain and the strain derived from river water that was considered the source of infection. Nevertheless, in each study, there was some discordance between the patient-derived strains and the river-derived strains [3, 5]. In this study, we found that the strain isolated from the patient and the strain isolated from the environment were derived from a phylogenetically close strain. This finding indicates that the patient was infected by this organism living in the environment in Japan located in a temperate zone.

**Table 3:** Summary of published *C. haemolyticum* infections

	Year of report	Country	Age	Comorbidity	Diagnosis	Therapy	Outcome	Reference number
1	2008	USA	Unknown	Unknown	Unknown	Unknown	Unknown	2
2	2013	Japan	26	None	Necrotizing fasciitis	(1) Ampicillin/sulbactam + minocycline (2) Ceftazidime (3) Ciprofloxacin + gentamicin (4) Ciprofloxacin	Survived	1
3	2014	Thailand	4	None	Proctocolitis	(1) Ceftriaxone (2) Ceftriaxone + ciprofloxacin + metronidazole	Survived	6
4	2015	Japan	69	Cerebral infarction, hypertension	Pneumonia	(1) Ampicillin/sulbactam (2) Meropenem (3) Piperacillin/tazobactam	Survived	5
5	2016	USA	11	Congenital heart disease, endocarditis	Bacteremia	(1) Ceftriaxone (2) Meropenem + gentamicin + sulfamethoxazole/trimethoprim (3) Ceftriaxone + levofloxacin	Survived	4
6	2020	Japan	70s	Hypertension, diabetes, benign prostatic hyperplasia	Pneumonia	(1) Meropenem + levofloxacin (2) Ceftazidime + levofloxacin (3) Levofloxacin	Survived	3
7	This study	Japan	73	Alcoholic hepatitis, hyperglycemia, hypertension	Meningitis	Meropenem + vancomycin	Died	–

It should be noted that this is the only fatal case among *C. haemolyticum* infection reported. This patient’s comorbidities could explain his poor outcome. Both alcoholic hepatitis and hyperglycemic status are related to immunosuppression and poor outcome of infectious diseases [12, 13].

It has been noted that *C. haemolyticum* may be misidentified as pigment-nonproducing *C. violaceum* or that it may fail to be identified when conventional identification systems that use biochemical properties or other systems, such as matrix-assisted laser desorption ionization-time of flight–mass spectrometry (MALDI-TOF–MS), are used for identification [1–5]. We experienced a similar situation in this study. *C. haemolyticum* has characteristics that are easy to distinguish from those of *C. violaceum*, such as a β-hemolytic ring on sheep blood agar (+), poor growth on MacConkey agar, oxidase (+), indole (−), mannose (−), citric acid (+), and developmental ability at 32 °C [1, 2, 11]. Because the name of this organism was not present in the database used by identification instruments, kits, and TOF–MS, it was labeled *C. violaceum* despite having these characteristics. Other hemolytic strains in the genus *Chromobacterium* have been isolated in recent years. For example, *C. paludis* shows weak hemolysis on 5% sheep blood agar after 4–5 days of incubation at 25 °C [10]. Although human infections by *C. paludis* have not been reported, attention should be given to misidentification with *C. haemolyticum*.

Regarding susceptibility testing, *C. haemolyticum* has been reported to be more resistant to β-lactams than *C. violaceum* [1, 2]. The strains detected in this study also showed high MIC values against penicillins and cephalosporins. Therefore, it should be noted that *C. haemolyticum* could be misidentified as *C. violaceum* by conventional methods, and it is important to identify *C. haemolyticum* appropriately. The findings of hemolysis on sheep blood agar and pigment production could distinguish *C. violaceum* and *C. haemolyticum*.

In summary, *C. haemolyticum* could be misidentified as *C. violaceum* by conventional identification methods and tends to be more resistant to β-lactams than *C. violaceum*. Pigment production and β-hemolysis on blood sheep agar can provide clues for the early identification of *C. haemolyticum*.

## Data Availability

All data are contained in the manuscript.

## Abbreviations

ANI: Average nucleotide identity
CFU: Colony-forming unit
MALDI-TOF–MS: Matrix-assisted laser desorption ionization-time of flight–mass spectrometry
MIC: Minimum inhibitory concentration
SNP: Single nucleotide polymorphism
